# Lung Cancer development and mortality in interstitial lung disease with and without connective tissue diseases: a five-year Nationwide population-based study

**DOI:** 10.1186/s12931-019-1094-y

**Published:** 2019-06-10

**Authors:** Won-Il Choi, Dong Yoon Lee, Hyun-Gi Choi, Choong Won Lee

**Affiliations:** 1 Department of Internal Medicine, Keimyung University Dongsan Hospital, 56 Dalseong-ro, Jung-gu, Daegu, 41931 Republic of Korea; 2 Department of Chemistry, Konkuk University, Seoul, 05029 Republic of Korea; 3 Department of Occupational and Environmental Medicine, Sungso Hospital, Andong, 99 Seodongmun-ro, Andong, Gyeongsangbuk-do 36690 Republic of Korea; 4 Department of Internal Medicine, Keimyung University Dongsan Hospital, 56 Dalseong-ro, Jung-gu, Daegu, 700–712 Republic of Korea

**Keywords:** Lung cancer, Connective tissue diseases, Interstitial lung disease, Mortality

## Abstract

**Background:**

Connective tissue disease associated with interstitial lung disease (CTD-ILD) and interstitial lung disease (ILD) alone have same pathological and imaging backgrounds. However, the differences between lung cancer development and the mortality risk between these two conditions are unclear. Incidence of primary lung cancer and all-cause mortality were studied between interstitial lung disease patients with and without connective tissue disease.

**Methods:**

Data were extracted from the Korean National Health Insurance Research Database in 2009. A total of 12,787 cases of ILD without idiopathic pulmonary fibrosis and 2491 cases of CTD-ILD were diagnosed in 2009. The cohort was followed up until June 30, 2014. Incident lung cancers and all-cause mortality were ascertained.

**Results:**

The overall incidence of lung cancer was 165.7 and 161.8 per 10,000 person-years in the CTD-ILD and ILD-only, respectively (rate ratio, 1.08; 95% confidence interval, 0.89–1.30). CTD-ILD patients in the 40–49 and 50–59 years old age groups had lung cancer incidence rates of 92.5 and 139.2, which were 2.0 and 1.7 times higher than those in the ILD-only, respectively. All-cause mortality was significantly higher in the CTD-ILD group compared to ILD-only group in patients aged 50–79 years. All-cause mortality of women in the 50–59, 60–69 and 70–79 age groups was 2.0, 1.8, and 1.4 times higher in the CTD-ILD group than in the ILD-only group, respectively.

**Conclusions:**

CTD-ILD patients aged < 60 years had a higher lung cancer incidence than ILD-only patients in the same age group. Furthermore, CTD-ILD patients aged 50–79 years had higher all-cause mortality than ILD-only patients in the same age group.

## Background

Connective tissue diseases (CTDs) can result in the development of pulmonary manifestations and considered as major organ involvement. CTD may be accompanied by interstitial lung disease (ILD) due to rheumatoid arthritis (RA) in 20–62% of cases [1–4], systemic lupus erythematosus in up to 70% of cases [5, 6], systemic sclerosis in 40–90% of cases [7, 8], and myositis in 20–66% of cases [9–11].

Various CTDs share similar lung pathologies [12]. The number of germinal centers associated with lymphocyte aggregation increases, and most inflammatory lesions in patients with CTD associated with interstitial lung disease (CTD-ILD) are distinct compared to those of patients with ILD alone (ILD-only) [13]. These findings suggest that diverse collagen vascular-related ILDs can be grouped into a single category termed CTD-ILD. CTD-ILD and ILD also share the same lung pathology spectrum [14]. In addition, certain forms of CTD-ILD also share genetic features with other interstitial lung abnormalities [15–17]. Although CTD-ILD with a nonspecific interstitial pneumonia (NSIP) pattern is the most prevalent form [18], no specific radiographic pattern identifies the disease [19].

Patients with CTD are also prone to developing lung cancer. Systemic sclerosis, dermatomyositis/polymyositis, rheumatoid arthritis, and a variety of CTDs have been associated with a high incidence of lung cancer [20–22]. Moreover, CTD-ILD is more often associated with lung cancer than CTD alone. Because patients with ILD alone also have a high lung cancer incidence [23, 24], controlling for ILD is important during evaluations of the lung cancer incidence in CTD-ILD patients.

To identify a meaningful number of patients with lung cancer from among those with orphan lung diseases (e.g., ILD), large data sets must be utilized, such as entire national insurance databases. Korea has only one health insurance system, the National Health Insurance (NHI) service. Inclusion of a large number of patients with CTD-ILD and ILD with follow-up data may allow investigation of the relationship between CTD-ILD and lung cancer according to age and sex after controlling for the effects of ILD alone.

Thus, this nationwide, 5-year, longitudinal, population-based study investigated the incidence of lung cancer and all-cause mortality among patients with CTD-ILD compared to those with ILD among patients without idiopathic pulmonary fibrosis (ILD-only) to help clarify the disease behavior.

## Methods

### Subjects

This closed longitudinal cohort study included data collected from the National Health Insurance (NHI) system based on the entire population. Since each citizen in Korea has a unique resident registration number, data duplication was avoided. The NHI service—the only public insurance system operated by the Ministry of Health and Welfare in Korea—is compulsory and covers the entire population, without exceptions for seasonal or part-time workers or unemployed persons [25]. The current study was approved by the institutional review board (IRB) at Dongsan Hospital, Keimyung University School of Medicine (IRB 2015–05-006). The IRB waived the requirement for informed consent.

### Case identification

According to the National Statistical Office of Korea, the mid-year population of people ≥40 years of age in 2009 was 22,280,691. Patients with ILD only were selected as control subjects. Patients with CTD-ILD or ILD only between January and December 2009 were enrolled. The International Classification of Diseases, 10th revision (ICD-10) codes were used as a key reference not only for disease diagnosis but also within the NHI database.

Specific codes for each CTD and IPF (J84.1A) have been implemented by the NHI service since (2009). Patients classified under autoimmune diseases including CTD, IPF, and lung cancer are eligible for a copayment reduction from the NHI; this is in line with the government’s improved policy regarding enhanced support for orphan diseases since 2009. Specific code for primary lung cancer (C34) has been implemented since 2005. When a patient is registered with CTD, IPF, or lung cancer, the physician should send the necessary eligibility documents to the NHI.

The cases and controls were identified between January and December 2009 and were followed up until June 2014. The diagnostic codes for primary lung cancer cases diagnosed before 2009 were maintained on the NHI database. New lung cancer cases were identified by counting new cases registered during the calendar year after excluding preexisting lung cancer. We counted newly developed lung cancer cases and death after 1 month of recruiting CTD-ILD and ILD-only cases to exclude cases simultaneously diagnosed as both ILD and lung cancer.

Death were identified using the NHI database. Deceased person is dropped off insurance coverage.

### Definitions of ILD-only and CTD-ILD

**Fig. 1:**
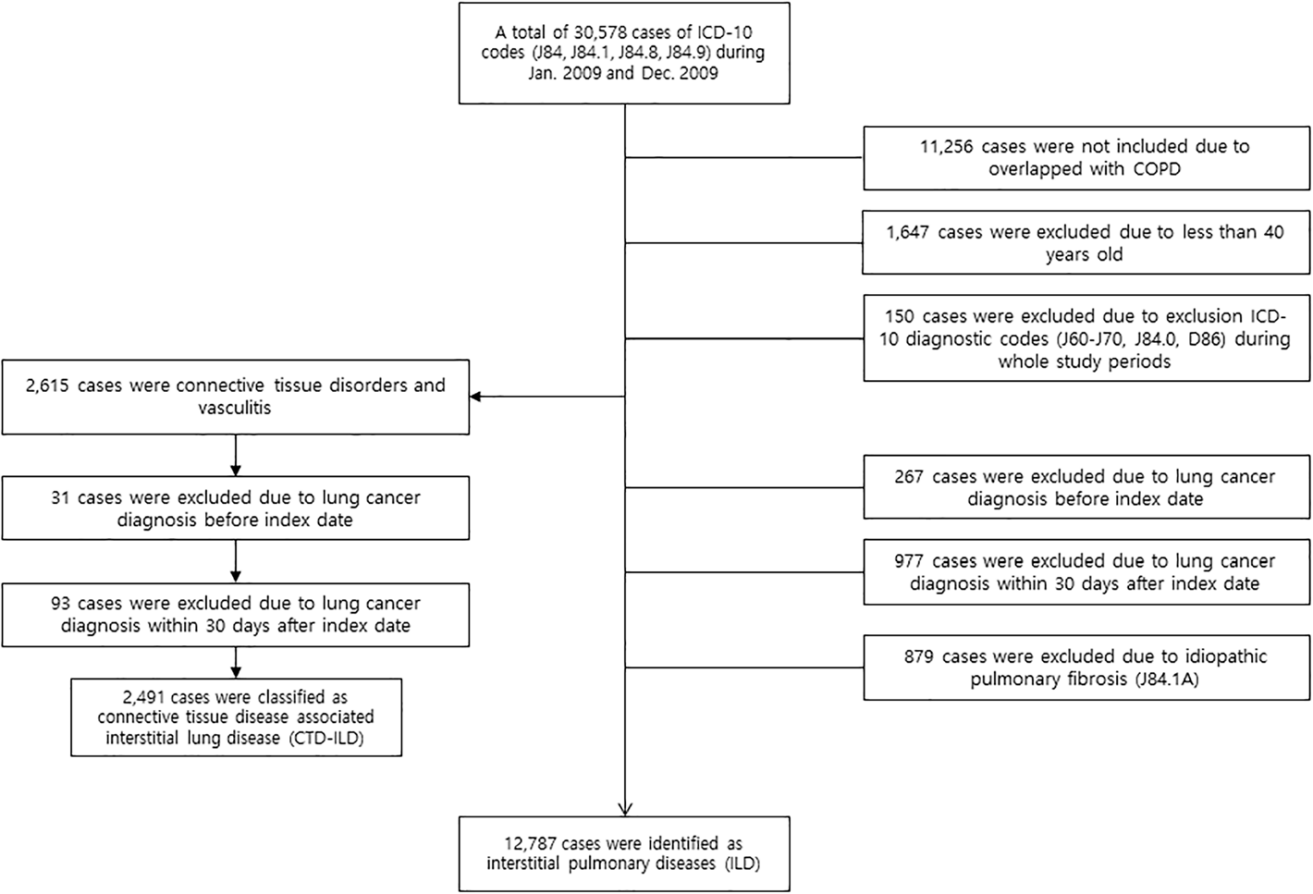
Study flowchart. Flowchart of patient selection for CTD-ILD and ILD-only. COPD, chronic obstructive pulmonary disease; CTD-ILD, connective tissue disease-associated interstitial lung disease; ICD, International Classification of Disease; ILD, interstitial lung disease

Based on the ICD-10 definitions, ILD-only was defined by code J84 for other ILD, excluding drug-induced ILD, interstitial emphysema, and lung diseases caused by external agents. In order to identify ILD without specific related diseases, connective tissue disease-associated ILD, hypersensitivity pneumonitis, sarcoidosis, and COPD were not included in the cohort. Code J84.x excluding J84.0, chronic pulmonary fibrosis due to inhalation of chemicals, gases, fumes, or vapors or that occurred following radiation therapy during the study period (Fig. 1).

The codes for identification of systemic CTD included M05 for rheumatoid arthritis, M07 for psoriatic and enteropathic arthropathies, M30 for polyarteritis, M31 for other necrotizing vasculopathies, M32 for systemic lupus erythematosus, M33 for dermatopolymyositis, M34 for systemic sclerosis, M35 for other systemic involvement of connective tissue, and M45 for ankylosing spondylitis. CTD-ILD was defined as any systemic CTD with J84 codes. In the ILD-only group, we excluded code J84.1A for idiopathic pulmonary fibrosis (IPF), which has been implemented since 2009, in much the same way as the new diagnostic criteria introduced in 2011 [26].

### Data verification

Every patient with CTD, lung cancer, and IPF sends a document to the NHI that aligns with the diagnostic criteria of each CTD. All CTD patients registered in the NHI. All patients with cancer are registered in a separate national cancer database maintained by the National Cancer Center (NCC) and strictly validated before final registration. If patients change their lung cancer diagnosis, they are later dropped off from the cancer code.

### Statistics

Age is reported using five-year units in the NHI data. We decided to divide the age groups by 10 years to account for the number of patients. The incidence rates of lung cancer per 10,000 person-years were calculated for patients with ILD with and without CTD according to the sex and age groups [27]. The incidence and mortality rates were compared as rate ratios (RRs) among different sex and age groups [28]. Kaplan-Meier curves were used to compare the development of lung cancer and all-cause mortality among patients with ILD with or without CTD. A sensitivity analysis was performed to assess the fragility of the results due to unmeasured confounding using the E-value methodology [29]. This E-value method estimates the minimum strength of association required between an unmeasured confounder and both the rate ratio of CTD-ILD to ILD-only and the risk of lung cancer development or mortality to overcome the statistically significant effect observed in a study where residual confounding is a potential problem (i.e., smoking). The reported *P*-values are two-sided, and those less than 0.05 are considered statistically significant. All analyses were performed using SPSS version 21 (IBM, Chicago, IL, USA) and the statistical software system R, version 3.5.2.

## Results

### Baseline demographics and follow-up duration

Women were predominant (71.1%) in the CTD-ILD group, whereas men were predominant (54.5%) in the ILD-only group. The median age was approximately 10 years less in patients with CTD-ILD compared to patients with ILD-only (Table 1). The median follow-up durations were 56.6 months (interquartile range [IQR], 48.3–64.9 months) and 54.8 months (IQR, 47.0–61.8 months) for the CTD-ILD and ILD-only groups, respectively.

**Table 1:** Demographics of patients with connective tissue disease-associated with interstitial lung disease (CTD-ILD) and interstitial lung disease (ILD)-only

Characteristic	CTD-ILD (*n* = 2491), n (%)	ILD-only, *n* = 12,787), n (%)
Sex		
Men	721 (28.9)	6969 (54.5)
Women	1770 (71.1)	5818 (45.5)
Age		
Median (Q1, Q3)	56 (51, 60)	65 (55, 67)
40–49	472 (18.9)	1667 (13.0)
50–59	685 (27.5)	2679 (21.0)
60–69	753 (30.2)	3535 (27.6)
70–79	493 (19.8)	3446 (26.9)
≥ 80	88 (3.5)	1460 (11.4)

Summarized based on the diagnosis at the top of the lists of International Statistical Classification of Diseases and Related Health Problems, 10th edition (ICD-10) codes of respiratory diseases

### Lung cancer development in the CTD-ILD and ILD-only

**Fig. 2:**
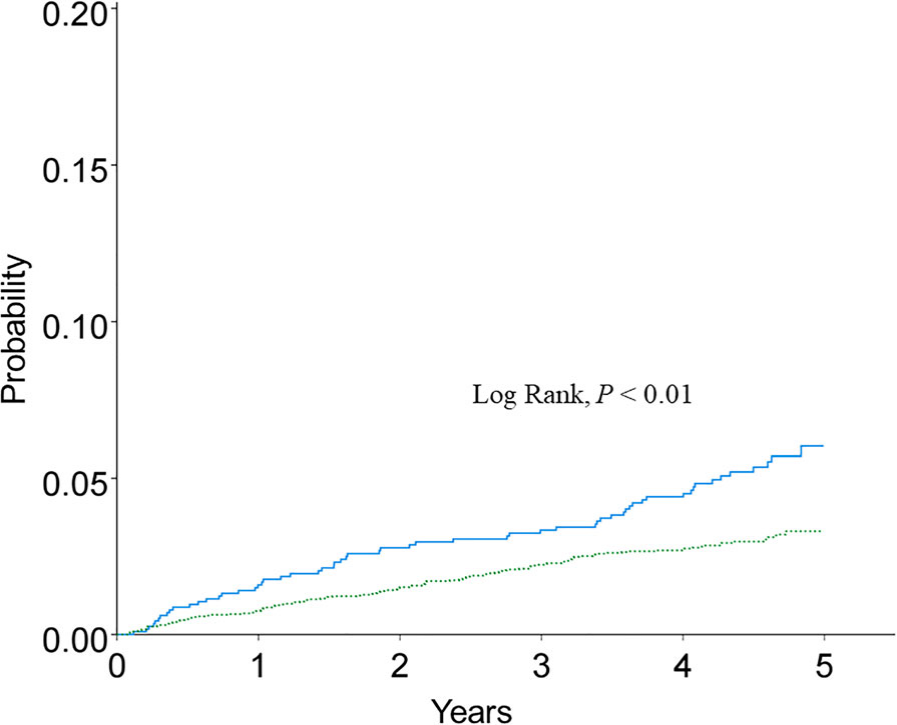
Kaplan-Meier curve for cumulative incidence of lung cancer below age 60 among the CTD-ILD (solid line) and ILD-only (dotted line). *CTD-ILD* connective tissue disease-associated interstitial lung disease, *ILD-only* interstitial lung disease

**Fig. 3:**
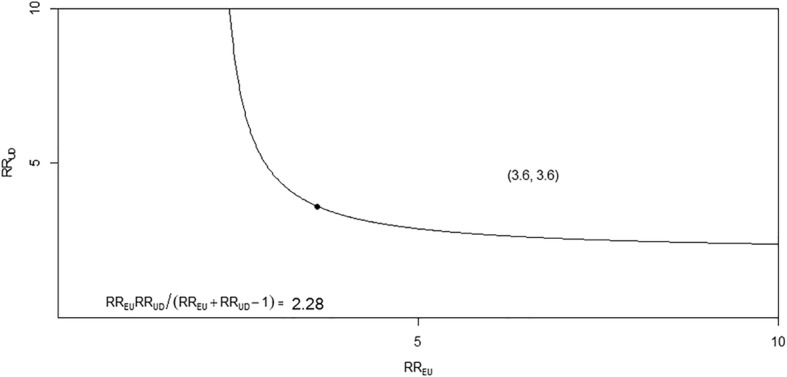
The minimum value required for the joint minimum strength of association of the risk ratio scale for an unmeasured confounder with the CTD-ILD and ILD-only groups to fully explain an observed treatment–outcome risk ratio of *RR* = 2.28 in the 40 to 49 years old age group. The E-value essentially sets the 2 parameters (*RR*_*UD*_ and *RR*_*EU*_) equal to each other to determine the required minimum for both values. The E-value estimate corresponds to the point (3.60, 3.60) in the 40 to 49 years old age group. RR, relative risk; E, exposure; U, unmeasured cofounder; D, outcome; *RR*_*EU*_, maximum risk ratio for any specific level of the unmeasured confounders comparing the lung cancer incidence between the CTD-ILD and ILD-only; *RR*_*UD*_, maximum risk ratio for the outcome when comparing any 2 categories of the unmeasured confounders; CTD-ILD, connective tissue disease-associated interstitial lung disease; ILD-only, interstitial lung disease

A total of 165.7 and 161.8 lung cancer cases per 10,000 person-years were included in the CTD-ILD and ILD-only groups, respectively (Table 2). We evaluated the lung cancer incidence between the two groups based on age (40 to 59 years and greater than 60 years). Lung cancer was more common in CTD-ILD than in the ILD-only patients for age group 40 to 59 years (Fig. 2 and Table 2). The E values for the differences in the lung cancer incidence between the CTD-ILD and ILD-only groups were 3.59 in the age group 40 to 49 years and 2.87 in the age group 50 to 59 years (Fig. 3).

**Table 2:** Lung cancer incidence per 10,000 person-years by age in patients with connective tissue disease-associated interstitial lung disease (CTD-ILD) and interstitial lung disease (ILD)-only

	CTD-ILD				ILD-only							
Age (years)	Population (n)	Lung cancer cases (n)	Person-years of follow-up	Incidence	Population (n)	Lung cancer cases (n)	Person-years of follow-up	Incidence	RR	95% CI	*P* value	E value
40–49	472	19	2054.3	92.5	1667	28	6924.3	40.4	2.28	1.20–4.24	0.01	3.60 (1.00-NA)
50–59	685	40	2873.7	139.2	2679	95	11,109.3	85.5	1.62	1.09–2.38	0.01	2.87 (1.43-NA)
60–69	753	59	2988.0	197.5	3535	220	14,041.9	156.7	1.26	0.92–1.68	0.13	
70–79	493	41	1768.7	231.8	3446	258	12,371.2	208.5	1.11	0.77–1.55	0.57	
> 80	88	6	271.4	221.1	1460	96	4232.2	226.8	0.97	0.34–2.20	1.00	
Total	2491	165	9956.1	165.7	12,787	697	48,679.0	143.2	1.15	0.97–1.37	0.10	

RR, rate ratio; CI, confidence interval; NA, not available; E value was calculated by VanderWeele and Ding method [29]

### Lung cancer development between the CTD-ILD and ILD-only according to sex and age

Among 40 to 49-years old men lung cancer incidence was more than three times higher in the CTD-ILD than in the ILD-only patients (Table 3). Among 50 to 59 years old women lung cancer incidence was more than two times higher in the CTD-ILD than in the ILD-only patients.

**Table 3:** Lung cancer incidence per 10,000 person-years by sex and age in patients with connective tissue disease-associated interstitial lung disease (CTD-ILD) and interstitial lung disease (ILD)-only

Men							Women					
Age (years)	Incidence CTD-ILD	Incidence ILD Only	RR	95% CI	*P* value	E value	Incidence CTD-ILD	Incidence ILD Only	RR	95% CI	*P* value	E value
40–49	161.7	48.8	3.23	1.11–9.39	0.03	5.91 (1.45-NA)	77.2	41.2	1.91	0.63–5.81	0.25	
50–59	201.8	107.2	1.83	0.97–3.46	0.06		119.3	40.8	2.81	1.23–6.42	0.01	5.06 (1.76-NA)
60–69	294.2	286.4	1.02	0.67–1.54	0.90		152.2	83.1	1.78	0.99–3.18	0.05	
70–79	352.3	288.8	1.20	0.75–1.93	0.43		170.6	129.1	1.31	0.74–2.32	0.35	
≥ 80	414.3	424.9	1.00	0.36–2.77	0.99		114.4	318.0	0.43	0.10–1.78	0.24	
Total	269.4	209.9	1.27	0.98–1.65	0.06		127.0	103.7	1.28	0.95–1.73	0.09	

*RR* Rate ratio, *CI* confidence interval, *NA* not available, E value was calculated by VanderWeele and Ding method [29]

### All-cause mortality in the CTD-ILD and ILD-only

**Fig. 4:**
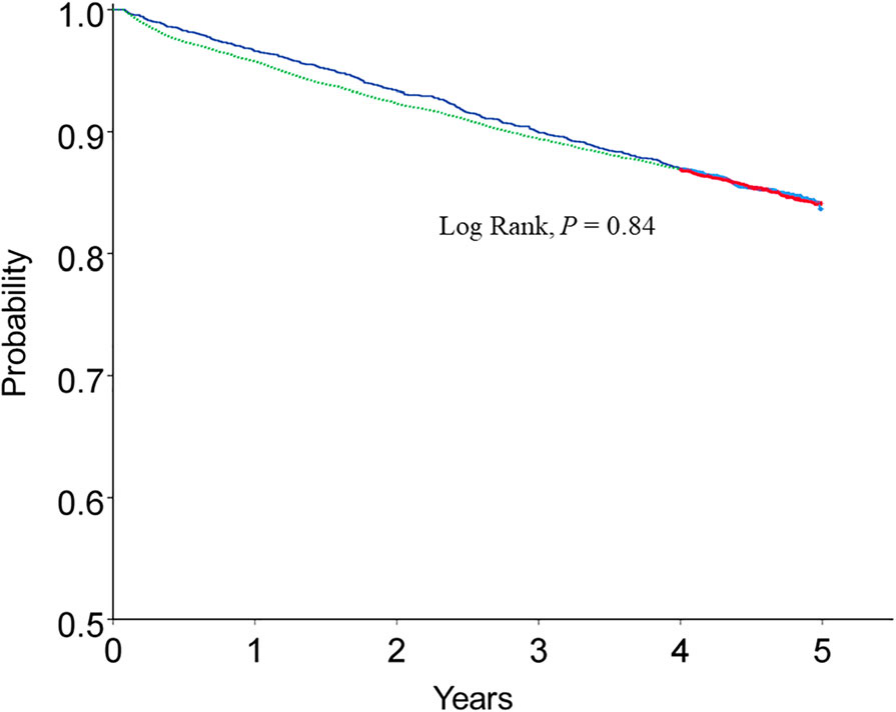
Kaplan-Meier curves for all-cause mortality for the CTD-ILD (solid line) and ILD-only (dotted line) patients. *CTD-ILD* connective tissue disease-associated interstitial lung disease; *ILD-only* interstitial lung disease

The all-cause mortality rates were 347.1 and 351.1 cases per 10,000 person-years in the CTD-ILD and ILD-only groups, respectively (Table 4). In patients aged between 50 and 79 years, all-cause mortality was significantly higher in the CTD-ILD than in the ILD-only group (Table 4). Mean survival was 4.55 (95% confidence interval [CI], 4.53–4.57) years and 4.59 (95% CI, 4.54–4.63) years in the CTD-ILD and ILD-only groups, respectively (Fig. 4).

**Table 4:** All-cause mortality per 10,000 person-years by age in patients with connective tissue disease-associated interstitial lung disease (CTD-ILD) and interstitial lung disease (ILD)-only

Age (years)	CTD-ILD				ILD-only							
	Population (n)	Deaths (n)	Person-years of follow-up	Mortality	Population (n)	Deaths (n)	Person-years of follow-up	Mortality	RR	95% CI	*P* value	E value
40–49	472	21	2161.2	97.2	1667	54	7386.2	73.1	1.32	0.76–2.23	0.33	
50–59	685	59	3032.5	194.6	2679	149	11,785.1	126.4	1.53	1.11–2.09	< 0.01	2.43 (1.45-NA)
60–69	753	120	3208.8	374.0	3535	429	15,021.0	285.6	1.30	1.06–1.60	0.01	1.92 (1.31-NA)
70–79	493	139	1990.8	698.2	3446	754	13,942.5	540.8	1.29	1.06–1.54	< 0.01	1.90 (1.31-NA)
> 80	88	33	323.5	1020.1	1460	493	5386.4	915.3	1.11	0.75–1.58	0.59	
total	2491	372	10,716.9	347.1	12,787	1879	53,521.4	351.1	0.98	0.88–1.10	0.86	

*RR* Rate ratio, *CI* confidence interval, *NA* not available, E value was calculated by VanderWeele and Ding method [29]

### All-cause mortality between patients of both sexes in the CTD-ILD and ILD-only

Among men, the all-cause mortality rates were 468.0 and 394.4 cases per 10,000 person-years in the CTD-ILD and ILD-only groups, respectively. Among women, the all-cause mortality rates were 299.8 and 304.5 cases per 10,000 person-years in the CTD-ILD and ILD-only groups, respectively. Among men 40 to 49-years, the all-cause mortality rate was more than 1.5 times higher in the CTD-ILD than in the ILD-only group (Table 5). Among women aged 50 to 69 years, the all-cause mortality rate was approximately 1.8 to 2 times higher in the CTD-ILD than in the ILD-only group (Table 6).

**Table 5:** All-cause mortality per 10,000 person-years by age in patients with connective tissue disease-associated interstitial lung disease (CTD-ILD) and interstitial lung disease (ILD)-only in men

Age (years)	CTD-ILD				ILD-only						
	Population (n)	Deaths (n)	Person-years of follow-up	Mortality	Population (n)	Deaths (n)	Person-years of follow-up	Mortality	RR	95% CI	*P* value
40–49	90	7	401.5	174.3	1002	38	4433.6	85.7	2.03	0.76–4.61	0.15
50–59	170	20	743.6	269.0	1559	107	6805.0	157.2	1.71	1.00–2.77	0.04
60–69	254	49	1054.7	464.6	2019	309	8418.7	367.0	1.26	0.97–1.71	0.15
70–79	172	52	686.9	757.0	1795	441	7176.5	614.5	1.23	0.90–1.64	0.18
> 80	35	13	126.3	1029.1	594	235	2091.1	1123.8	0.91	0.48–1.59	0.89
total	721	141	3013.0	468.0	6969	1130	28,925.1	390.7	1.19	0.99–1.42	0.05

RR, Rate ratio; CI, confidence interval

**Table 6:** All-cause mortality per 10,000 person-years by age in patients with connective tissue disease-associated interstitial lung disease (CTD-ILD) and interstitial lung disease (ILD)-only in women

Age (years)	CTD-ILD				ILD-only							
	Population (n)	Deaths (n)	Person-years of follow-up	Mortality	Population (n)	Deaths (n)	Person-years of follow-up	Mortality	RR	95% CI	*P* value	E value
40–49	382	14	1759.9	79.5	665	16	2952.5	54.2	1.46	0.66–3.20	0.38	
50–59	515	39	2289.4	170.4	1120	42	4980.1	84.3	2.01	1.27–3.20	< 0.01	3.43 (1.85-NA)
60–69	499	71	2154.8	329.5	1516	120	6602.2	181.8	1.81	1.33–2.45	< 0.01	3.02 (1.99-NA)
70–79	321	87	1304.2	667.1	1651	313	6766.0	462.6	1.44	1.12–1.83	< 0.01	2.23 (1.48-NA)
> 80	53	20	197.2	1014.2	866	258	3295.2	783.0	1.29	0.77–2.04	0.32	
total	1770	231	7705.7	299.8	5818	749	24,596.3	304.5	0.98	0.84–1.14	0.86	

*RR* Rate ratio, *CI*, confidence interval, *NA* not available, E value was calculated by VanderWeele and Ding method [29]

## Discussion

An increased lung cancer incidence in CTD-ILD patients compared to that in ILD-only patients was observed among those aged < 60 years. The lung cancer incidence rate ratios in men and women were 27 and 28%, respectively, in the CTD-ILD patients, which were higher than those with ILD-only. In 40 to 49 years old men lung cancer risk was 3.2 times higher for CTD-ILD patients than for those with ILD-only**.** The lung cancer risk for women in the 50 to 59 years age group was 2.8 times higher for CTD-ILD patients than for ILD-only patients. Although we could not exclude unmeasured confounders to eliminate the possibility of spurious results in these age groups, this result suggested that the mechanisms underlying enhanced lung cancer development in CTD-ILD patients involved a younger age for both men and women.

We previously reported the epidemiology of lung cancer in CTD-ILD, ILD, and idiopathic pulmonary fibrosis (IPF) patients [23, 30]. However, the number of lung cancer patients was small, and the data used to compare the lung cancer prevalence between the CTD-ILD and ILD-only groups were unstable [30]. Therefore, the previous study did not clearly show a difference in the lung cancer prevalence between the CTD-ILD and ILD-only. However, the other study demonstrated the lung cancer risk for IPF patients was significantly higher than ILD patients [23]. The present study included a whole national population that was followed for up to 5 years, which might have helped detect a significant difference in the lung cancer incidence between the CTD-ILD and ILD-only patients.

The incidence of lung cancer is high in men with RA [31–33]. In line with these studies, the present study shows that the lung cancer incidence was approximately two times higher in ILD irrespective of gender.

One objective of this study was to estimate the contribution of CTD to lung cancer development. In the younger age group (40 to 49 years), the lung cancer risk was 2.3 times higher in the CTD-ILD than in the ILD-only group, but the risk decreases with age. This phenomenon is commonly observed in both men and women. Based on these results, the following hypotheses can be considered. First, lung cancer development potential due to autoimmune responses is reduced as the patient ages. The second hypothesis is that autoimmunity combined with other factors contributes to an increased risk of lung cancer development at a young age. Third, the development of autoimmune lung disease related cancer may overlap with other powerful lung cancer-causing factors, such as the aging process; thus, lung cancer risk related to autoimmunity can be neglected.

The presence of fibrosing interstitial pneumonia in RA patients is associated with a lower survival rate than that of RA alone [34], and CTD-ILD has a poorer prognosis than idiopathic interstitial pneumonia [19]. ILD-only also revealed higher mortality than non-ILD control [35]. In contrast, several studies have shown opposite or neutral results in relation to survival [36–40]. In this population-based closed cohort study, aged between 50 to 79 years, mortality was higher in the CTD-ILD than the ILD-only group, especially for women.

### Study limitations

We could not control for the confounding effects of unmeasured smoking on the development of lung cancer, because the NHI database did not include smoking history data. ILD carries an increased risk of lung cancer, even after correcting for or considering the smoking status [30, 41]. The odds ratio of smoking for lung cancer development in CTD-ILD patients was reported to be 1.2 [42]. The use of immunosuppressants for CTD-ILD may also increase the incidence of lung cancer [42]. Although we also did not control for the confounding effects of immunosuppressants in this study, immunosuppressants are tightly related to de novo malignancy [43, 44]. In this study, because no general population control without ILD was included, we could not measure the true magnitude of the effect of ILD on lung cancer development.

We identified CTD-ILD based on the ICD-10 code. However, no specific ICD-10 code is available for CTD-ILD, and no clear consensus diagnosis exists concerning what constitutes CTD-ILD based on the ICD-10 code. Additionally, the ICD-10 code does not include histological findings. The patient data are anonymized, which makes it impossible to trace information back to a medical record. Therefore, we did not perform further analyses according to lung cancer histology.

We excluded patients with a diagnosis of lung cancer before and 1 month after the index date. However, patients with lung cancer could be miscounted due to the occurrence of new cases in the early period of the cohort. Additionally, other risk factors, such as pulmonary function and high-resolution computed tomography (CT) findings for ILD, could not be evaluated, because the NHI primarily included medical claims.

## Conclusions

We observed increased lung cancer incidence in younger (less than 60 years old) patients with CTD-ILD when compared to ILD-only. In addition, we found that all-cause mortality rate was higher in older CTD-ILD patients (50 to 79 years old) than in those ILD-only, especially in women. These results imply that a physician may pay more attention to lung cancer screening and management strategies for CTD-ILD patients.

## Data Availability

The datasets supporting the conclusion of this article is not available due to National Health Insurance data management policy.

## Abbreviations

CT: Computed tomography
CTD: Connective tissue disease
ICD: International classification of disease
ILD: Interstitial lung disease
IPF: Idiopathic pulmonary fibrosis
NCC: National cancer center
NHI: National health insurance
RA: Rheumatoid arthritis
